# Integration of transcriptome and proteome profiles in glioblastoma: looking for the missing link

**DOI:** 10.1186/s12867-018-0115-6

**Published:** 2018-11-21

**Authors:** Jean-Michel Lemée, Anne Clavreul, Marc Aubry, Emmanuelle Com, Marie de Tayrac, Jean Mosser, Philippe Menei

**Affiliations:** 1 Department of Neurosurgery, CHU Angers, University Hospital of Angers, 4, Rue Larrey, 49933 Angers Cedex 09, France; 2 CRCINA, INSERM, Université de Nantes, Université d’Angers, Angers, France; 3 UEB, UMS 3480 Biosit, Faculté de Médecine, Université Rennes 1, Rennes, France; 4 Plate-forme Génomique Santé Biosit, Université Rennes 1, Rennes, France; 5 Inserm U1085 IRSET, Université de Rennes 1, Rennes, France; 6 Protim, Université de Rennes 1, Rennes, France; 7 Service de Génétique Moléculaire et Génomique, CHU Rennes, Rennes, France; 8 CNRS, UMR 6290, Institut de Génétique et Développement de Rennes (IGdR), Rennes, France

**Keywords:** Glioblastoma, Molecular biology, Transcriptomics, Proteomics, SYN1, NEFL, TOPORS

## Abstract

**Background:**

Glioblastoma (GB) is the most common and aggressive tumor of the brain. Genotype-based approaches and independent analyses of the transcriptome or the proteome have led to progress in understanding the underlying biology of GB. Joint transcriptome and proteome profiling may reveal new biological insights, and identify pathogenic mechanisms or therapeutic targets for GB therapy. We present a comparison of transcriptome and proteome data from five GB biopsies (TZ) vs their corresponding peritumoral brain zone (PBZ). Omic analyses were performed using RNA microarray chips and the isotope-coded protein label method (ICPL).

**Results:**

As described in other cancers, we found a poor correlation between transcriptome and proteome data in GB. We observed only two commonly deregulated mRNAs/proteins (neurofilament light polypeptide and synapsin 1) and 12 altered biological processes; they are related to cell communication, synaptic transmission and nervous system processes. This poor correlation may be a consequence of the techniques used to produce the omic profiles, the intrinsic properties of mRNA and proteins and/or of cancer- or GB-specific phenomena. Of interest, the analysis of the transcription factor binding sites present upstream from the open reading frames of all altered proteins identified by ICPL method shows a common binding site for the topoisomerase I and p53-binding protein TOPORS. Its expression was observed in 7/11 TZ samples and not in PBZ. Some findings suggest that TOPORS may function as a tumor suppressor; its implication in gliomagenesis should be examined in future studies.

**Conclusions:**

In this study, we showed a low correlation between transcriptome and proteome data for GB samples as described in other cancer tissues. We observed that NEFL, SYN1 and 12 biological processes were deregulated in both the transcriptome and proteome data. It will be important to analyze more specifically these processes and these two proteins to allow the identification of new theranostic markers or potential therapeutic targets for GB.

**Electronic supplementary material:**

The online version of this article (10.1186/s12867-018-0115-6) contains supplementary material, which is available to authorized users.

## Background

Glioblastoma (GB) is the most common and aggressive primary tumor in the adult brain. Despite years of research and numerous clinical trials, survival remains poor [1]. Progresses have been made in understanding the underlying biology of GB thank to work involving genotype-based approaches and proteome analyses. The cancer genome atlas (TCGA) analysis identified genetic events that appear to be important in human GBs, including (i) deregulation of growth factor signaling via amplification and mutational activation of receptor tyrosine kinase (RTK) genes; (ii) activation of the phosphatidyl inositol 3-kinase pathway; and (iii) inactivation of the p53 and retinoblastoma tumor suppressor pathways [2]. Genome-wide profiling studies have highlighted the existence of molecular subtypes of GB with distinct biological features and clinical correlates [3–5]. For example, Verhaak et al. [3] described four subtypes of GB: proneural, neural, classical and mesenchymal characterized by abnormalities in PDGFRA, IDH1, EGFR and NF1. However, the definition of a Verhaak subtype for a whole tumor has been questioned because GBs are very heterogeneous tumors and recent studies showed that different samples from the same tumor can be of different Verhaak subtypes [6, 7]. Proteome profiling of human GB samples also revealed a protein cluster (Huntingtin, HNF4α, c-Myc and 14-3-3ζ) that is differentially expressed in GB and might also serve as a diagnostic marker [8, 9]. The methylation status of *MGMT* has also been identified through omic analyses: this feature predicts sensitivity to temozolomide, an alkylating agent that is the current standard treatment for GB patients [10]. Another identified biomarker is the isocitrate dehydrogenase 1 (IDH1) mutation, that has been identified and has diagnostic applications as it helps in distinguishing primary from secondary GB [2].

Until recently, the behavior of GB has been studied through independent analyses of the transcriptome or of the proteome [11–16]. Joint transcriptome and proteome profiling may reveal new biological insight, and identify pathogenic mechanisms or therapeutic targets for GB therapy.

We report the analysis of GB biopsies from five patients involving RNA microarray and isotope-coded protein label (ICPL) technologies, part of the Grand Ouest Glioma Project, a translational project aiming to study the intratumoral heterogeneity in GB [11, 12, 15–20]. The transcriptome and the proteome of the GB tumor zone (TZ) were defined by comparison with the corresponding peritumoral brain zone (PBZ). The integrated transcriptome and proteome analysis was based on the four different approaches described by Haider and Pal [21]: (1) intersection of transcriptome and proteome data, (2) identification of the common biological processes altered in the two datasets (3) identification of the common functional pathways altered in the two datasets, (4) topological network methods, with the analysis of the transcription factor binding sites (TFBSs) present upstream from the open reading frames of the altered proteins identified by ICPL method.

## Methods

### Patient recruitment

The entire project was approved by the local institutional review board (CPP Ouest II) and the Direction Générale de la Santé (DGS). All patients included in this study were diagnosed for de novo GB (WHO 2007 classification) by a central committee of neuropathologists and gave their written informed consent prior to their enrolment. Five patients (Table 1) with both proteome and transcriptome analysis of their TZ and PBZ tissues were selected from the databank of the “Grand Ouest Glioma Project”. More detailed information on tissue samples characteristics can be found in our previous publications [11, 15, 16].

**Table 1:** Description of patients’ characteristics

Patient ID	Age	Sex	Histology	GB variant (Verhaak)
GB-03	68	F	GB	Mesenchymal
GB-10	50	M	GB	Neural
GB-16	73	M	GB	Proneural
GB-25	61	F	GB	Proneural
GB-26	68	M	GBO	Neural

GBO, GB with an oligodendroglial component

### GB and control brain sampling

For each patient, image-guided neuronavigation was used during pre-surgical planning to define TZ and PBZ samplings sites. The TZ sample (volume around 1 cm^3^) was then collected in the contrast-enhanced area of the tumor by computer-assisted, image-guided brain biopsies before the surgical resection of the tumor (Brainlab^®^, La Défense, France). Control brain samples were taken from the PBZ to be used as control samples for transcriptomic and proteomic analyses in radiologically normal, non-enhancing brain at least 1 cm from the contrast enhancing tumor. All the PBZ showed minimal genomic alteration (< 1%) and did not show tumor cell infiltration on histopathological analysis except for the PBZ of GB-10 [15].

Samples were transferred to the Department of Pathology of the University hospital of Angers, France, for pathological diagnostic, and to transcriptomic and proteomic platforms in the University hospital of Rennes, France for molecular analyses.

### Transcriptome analysis

Transcriptome analyses of the tumor samples were performed as previously described in one of our previous publication on the transcriptomic platform Biosit, Rennes, France [16, 22]. In brief, total RNA was isolated from the GB samples using the NucleoSpin RNAII kit (Macherey–Nagel, Hoerdt, France) and RNA integrity (RNA integrity NC8) was assessed with an Agilent 2100 bioanalyzer (Agilent Technologies, Santa Clara, CA, USA). Extraction of RNA microarray data was performed with an Agilent Whole Human Genome 4 × 44 K Microarray 15 Kit (Agilent technologies), according to the manufacturer’s recommendations.

Raw RNA data were then log2-transformed and normalized (quantile normalization and baseline transformation) using *R v3.1.0*. (http://www.r-project.org). For the transcriptomic profile identification, we used a non-parametric rank product method to account for hybridization bias, allowing the identification up- or down-regulated genes in pooled GB tumor tissue by comparison to the pooled peritumoral brain samples, using the *RankProd* R package. RNAs were considered significantly differentially expressed if the false detection rate (FDR) was below 0.05 and the absolute fold-change between pooled data from TZ vs. PBZ was greater than 2.

### Proteome analysis

The protocol for proteome analyses has already been described in our previous publications [11, 12]. TZ samples were analyzed using ICPL, that allows a high-throughput identification and quantification of a sample’s protein profile [23, 24]. Intact proteins were labeled with isotopic derivatives of nicotinic acid of different molecular weight, then subject to gel liquid chromatography and tandem mass spectrometry with an Esquire HCT Ultra PTM Discovery mass spectrometer, to identify and quantify proteins.

Peptides were identified by querying the human Swiss-Prot database with the Mascot search engine (v.2.2.07) applying a score above the identity threshold and a FDR < 1%. Differentially expressed proteins were identified in the TZ by comparison to the PBZ samples with a threshold > 1.41 for up-regulated proteins and < 0.71 for down-regulated proteins, which is above the calculated technical variation of the method [11].

### Comparison of transcriptome and proteome data

We used four different methods to compare the results from the transcriptome and proteome analyses of the GB tumor tissues:

1. We performed a direct comparison of the intersection of transcripts and proteins found to be deregulated between TZ samples and their corresponding PBZ, to identify the overlap of direct features between transcriptome and proteome data. The comparison of the results from the transcriptome and proteome analyses of the GB tumor tissues was performed on pooled patient data and on paired patient to reduce the uncertainty due to variability between the patients.
2. We conducted an analysis of the biological and functional processes found to be altered in transcriptome and proteome data using DavidGenes (http://david.abcc.ncifcrf.gov). The probability of alteration of the biological process was calculated using one-sided Fisher exact P-value and the False Discovery Rate (FDR) was calculated using one-sided Fisher exact P-value corrected for multiple comparisons. Biological and functional processes were considered significantly altered in each dataset with P and FDR < 0.05.
3. The functional pathways identified by both transcriptome and proteome data as being altered were identified using KEGG database (http://www.genome.jp/kegg/pathway.html). Functional pathways were considered significantly altered in each dataset with P < 0.05 corrected for multiple comparisons using Benjamini–Hochberg method, and a fold-change > 2.
4. We looked for the presence of direct edges between transcripts and proteins with the identification of TFBSs in the regulatory region upstream from the open reading frames of the proteins identified as deregulated in GB. The main objective of this analysis was to identify factors that may bind to (and increase the expression of) the DNA encoding proteins identified in proteome analysis as being over-expressed. The Multi-genome Analysis of Positions and Patterns of Elements of Regulation search engine was used, running the set of proteins found to be deregulated in GB in our study (MAPPER_2_, http://genome.ufl.edu/mapper/#se). A graphical summary of the analysis techniques is available in the Additional file 1: Figure S1.

### Western blot analysis

TZ and PBZ samples (n = 11) were lysed in RIPA buffer containing a protease inhibitor cocktail and PMSF (Fisher Scientific, Illkirch, France) at 4 °C for 30 min. The lysates were clarified by centrifugation at 14,000*g* at 4 °C for 30 min. Protein concentrations were determined using the Pierce™ BCA protein assay kit (Fisher Scientific) with BSA as the standard, and equal samples of proteins (10 μg/lane) from the samples were resolved on a 7.5% SDS–polyacrylamide gel. The proteins were then electrotransferred onto PVDF membranes. After blocking in TBS blocking buffer (Fisher Scientific) at 4 °C overnight, blots were incubated with the respective primary antibodies [anti-actin (Merk Millipore, Guyancourt, France), anti-neurofilament light polypeptide (NEFL), anti-synapsin 1 (SYN1) and anti-topoisomerase I binding, arginine/serine-rich, E3 ubiquitin protein ligase (TOPORS) (CliniSciences, Nanterre, France)] for 2 h at room temperature. Horseradish peroxidase-conjugated secondary antibodies were then used and visualized with an enhanced chemoluminesence (ECL) reagent and the LAS4000 digital imaging system (Fisher Scientific).

## Results

### Direct comparison of deregulated RNA and proteins

The transcriptome pooled analysis with 41,000 probes of the five TZ vs PBZ samples identified 478 mRNAs differentially expressed between TZ samples and their corresponding PBZ samples. A total of 437 non-redundant genes were identified; 101 genes were over-expressed in the florid TZ, and 300 genes were under-expressed (list of differentially expressed probes in Additional file 2: Table S1). Proteome analysis identified 584 non-redundant proteins, and 259 were quantified: 31 proteins were found to be up-regulated in the TZ in at least 3/5 patients (Full proteome data available in [11]).

**Fig. 1:**
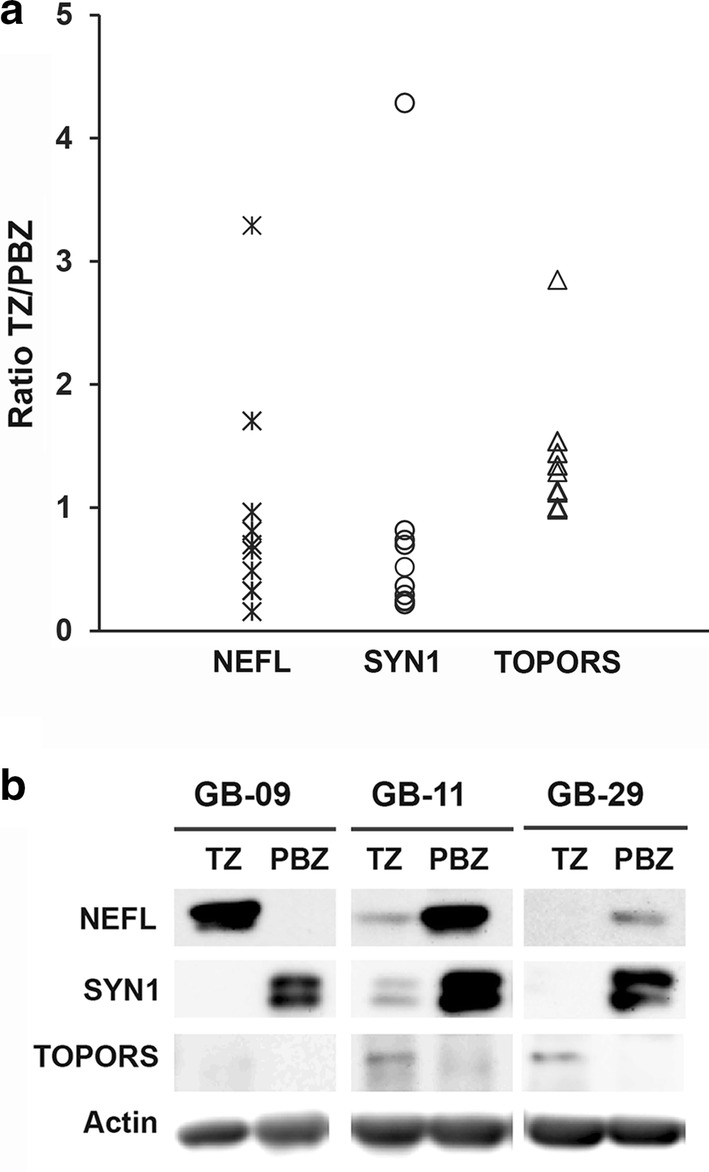
Analysis of NEFL, SYN1 and TOPORS expression in TZ biopsies and their corresponding PBZ (n = 11). **a** Distribution of densitometry data obtained for NEFL, SYN1 and TOPORS in 11 GB patients. Data are presented as TZ/PBZ ratios. **b** Example of western blot showing the expression of NEFL, SYN1, TOPORS and actin in TZ and PBZ samples

The intersection between transcriptome and proteome data consisted of two genes: that for the NEFL and that for SYN1. They did not show the same deregulation in the two omic analyses: transcriptome analysis indicated that they are under-expressed in TZ whereas proteome analysis indicated that they are over-expressed. The Western blot analysis of expression of NEFL and SYN1 in a larger cohort of TZ samples and their corresponding PBZ (n = 11) confirmed the transcriptomic results; in most cases, an under-expression of NEFL and SYN1 proteins was observed in the TZ (9/11 for NEFL and 10/11 for SYN1) (Fig. 1).

We also performed paired patient-specific comparisons to reduce the uncertainty due to variability between the patients. We observed similar trends with the pooled data TZ vs. PBZ comparisons with a low correlation rate (about 24%) between transcriptomic and proteomic data (Table 2).

**Table 2:** Patients-paired comparison of transcriptomic and proteomic data

Patient ID	GB-03	GB-10	GB-16	GB-25	GB-26
Number of proteins quantified	105	135	114	48	116
Number of altered proteins	50	53	93	76	53
Transcriptomic correlation	26%	14%	27%	25%	26%

Patients-paired comparison of transcriptomic and proteomic data was based on the significantly deregulated transcripts (P < 0.05 and fold-change > 2) and proteins between TZ and PBZ. The correlation rate (%) between the two modalities is indicated

### Comparison of altered biological processes

The transcriptomic data indicated that 149 biological processes were altered in the tumor samples, and the proteome analysis indicated that 23 biological processes were altered (list in Additional file 3: Table S2). Twelve biological processes were found to be altered in both datasets, all repressed in the transcriptome analysis but enriched in the proteome analysis, except for the “regulation of biological quality” process, which was enriched in both datasets (Table 3).

**Table 3:** Biological processes found to be altered in both transcriptome and proteome datasets

Gene ontology	Transcriptome					Proteome				
	Total genes	Diff. genes	P-value	FDR (%)	Enr.	Total genes	Diff. genes	P-value	FDR (%)	Enr.
GO:0003008—*System_process*	840	40	< 0.001	< 0.01	–	27	9	0.01	0.37	2.1
GO:0007267—*Cell-cell_signaling*	612	38	< 0.001	< 0.01	–	23	8	0.01	0.40	2.2
GO:0007268—*Synaptic_transmission*	297	31	< 0.001	< 0.01	–	18	7	0.01	0.58	2.4
GO:0019226—*Transmission_of_nerve_impulse*	326	33	< 0.001	< 0.01	–	20	7	0.02	0.43	2.2
GO:0023046—*Signaling_process*	1975	63	< 0.001	< 0.01	–	41	13	0.002	0.25	2
GO:0023060—*Signal_transmission*	1973	63	< 0.001	< 0.01	–	41	13	0.002	0.25	2
GO:0050877—*Neurological_system_process*	541	35	< 0.001	< 0.01	–	21	7	0.03	0.39	2.1
GO:0007154—*Cell_communication*	1174	41	0.001	< 0.01	–	32	9	0.04	0.36	1.8
GO:0007269—*Neurotransmitter_secretion*	50	8	0.002	< 0.01	–	5	3	0.03	0.45	3.8
GO:0023052—*Signaling*	2871	71	0.003	< 0.01	–	53	13	0.03	0.46	1.5
GO:0044057—*Regulation_of_system_process*	135	8	0.05	0.04	–	5	3	0.03	0.45	3.8
GO:0065008—*Regulation_of_biological_quality*	1443	21	0.05	0.02	2	49	12	0.05	0.35	1.5

P-value corrected for multiple comparisons

*Diff. genes* differentially expressed genes, *Enr*. enrichment; *FDR* False Discovery Rate calculated with one-sided Fisher exact

### Comparison of altered functional pathways

The analysis of altered functional pathways using the KEGG database found eight upregulated functional pathways in transcriptome analysis and two upregulated pathways in proteome analysis. There was no significant overlap between transcriptome and proteome analyses (Table 4).

**Table 4:** Functional pathways altered in transcriptome and proteome analyses in KEGG database

Pathway	Genes	Official gene symbol	Fold change	P-value	TNC
***Transcriptomic***					
hsa04080: *neuroactive ligand–receptor interaction*	18	GABRG1, GABRD, GABRG2, GABRA2, GABRA1, CCKBR, GRIN1, OXTR, GABBR2, GRM1, GRIN2C, SSTR1, PRSS2, PRSS3, ADRA1B, HTR5A, F2R, HTR2A	3.69	< 0.01	3103
hsa04020: *calcium signaling pathway*	14	CCKBR, GRIN1, OXTR, ITPKA, GRM1, ATP2B3, GRIN2C, ADRA1B, RYR2, CAMK2B, CAMK2A, HTR5A, F2R, HTR2A	4.17	< 0.01	3111
hsa04512: *ECM-receptor interaction*	10	IBSP, COL4A2, COL4A1, CD44, TNC, COL3A1, COL1A2, SV2B, COL1A1, FN1	6.24	< 0.01	3125
hsa00910: *nitrogen metabolism*	4	GLS2, CA9, CA12, GLS	9.12	0.19	3076
hsa04720: *long-term potentiation*	6	GRIN2C, PPP1R1A, GRIN1, CAMK2B, CAMK2A, GRM1	4.63	0.15	3157
hsa05014: *amyotrophic lateral sclerosis*	5	SLC1A2, GRIN2C, GRIN1, NEFH, NEFL	4.95	0.24	3125
hsa04510: *focal adhesion*	9	IBSP, PAK6, COL4A2, COL4A1, TNC, COL3A1, COL1A2, COL1A1, FN1	2.35	0.38	3103
hsa00471: *d*-*glutamine and d*-*glutamate metabolism*	2	GLS2, GLS	26.21	0.59	3333
***Proteomic***					
hsa05130: *pathogenic* *Escherichia coli* *infection*	5	ACTB, TUBB2A, TUBB2C, TUBB4, YWHAZ	19.39	0.00	30
hsa00010: *glycolysis/gluconeogenesis*	4	ALDOA, PGAM1, HK1, ENO1	14.74	0.04	30

*TNC* total number of components in each pathway

### Identification of TFBSs

We identified six specific TFBSs present upstream from the open reading frames of the altered proteins identified (Table 5, Additional file 4: Table S3). Of interest, a binding site for the RING finger protein TOPORS was present upstream from the open reading frames of all altered proteins. TOPORS was not deregulated at the mRNA level in the transcriptomic analysis (Additional file 4: Table S3) and it was not identified in the proteome analysis. We performed the expression of TOPORS by Western blot analysis on 11 TZ biopsies and their corresponding PBZ. We didn’t observe TOPORS expression in PBZ while 7/11 TZ samples expressed this protein with a TZ/PBZ ratio of 1.14–2.85 (Fig. 1).

**Table 5:** Transcription factor binding sites (TFBSs) identified upstream from the open reading frames encoding the deregulated proteins

TFBS	Proteins with TFBS	P-value
bZIP911	MBP	0.05
NIL	SYN1	< 0.01
PPARG	TUBB4	0.01
PPARG-RXRA	GDI1, MBP, TUBB4	0.01–0.04
RSRFC4	SYN1	0.03
TOPORS/LUN-1	All	< 0.01–0.05

P-value: probability of the presence of the TFBS upstream from the open reading frame of the protein, corrected for multiple testing using the Benjamini–Hochberg method

## Discussion

Although the comparison of transcriptomic and proteomic profiles has been done in several cancers, this study is the first of which we are aware to compare transcriptome and proteome data in a same cohort of GBs. Haider and Pal [21] reviewed the existing major approaches for joint analysis of transcriptome and proteome data. As recommended by this review, we directly compared the deregulated proteins and mRNAs, and then compared the functional processes and regulators identified in these transcriptome and proteome data sets.

There were few common features in the transcriptome and proteome data: they were the deregulation of two mRNAs/proteins (NEFL and SYN1) and 12 biological processes; they are related to cell communication, synaptic transmission and nervous system processes. These findings are consistent with previous reports [11, 16, 38]. No biological processes linked to tumorigenesis, for example cell cycle regulation, cell metabolism or cell motility, were found in both transcriptome and proteome analysis to be altered in GB. Some such processes were found in one dataset to be altered in GB, for example d-glutamine metabolism in the transcriptome and glycolysis/glyconeogenesis in the proteome [39, 40]. We observed the “pathogenic *Escherichia coli* infection” enrichment in GB through the proteome analysis. Its relation with the GB is presently unknown. Considering the involvement of genes in multiple biological processes, this enrichment could be an artefact. However, recent studies highlight the role of microbiota in gastric and breast cancer developments and may be present in GB [25, 26]. Further studies are needed to reply to this observation.

Although NEFL, SYN1 and twelve biological processes were in common between the two omic analyses, they did not show the same deregulation (except for the “regulation of biological quality” process): transcriptome analysis indicated that they are under-expressed in TZ whereas proteome analysis indicated that they are over-expressed. Mismatches of this type between such pairs of datasets has already been described [21, 25, 26]; there are several possible explanations including translation of mRNAs being up-regulated by RNA binding proteins and/or down-regulation of miRNA targeting these mRNAs. Also, the half-lives of mRNAs are very much shorter than those of proteins and protein stability may be affected by post-translational modifications like phosphorylation, acetylation and glycosylation [21, 27–29]. Note that Western blot analysis on more TZ/PBZ samples from other GB patients confirmed the deregulation of NEFL and SYN1 at the protein level but in most cases, an under-expression of these proteins was observed in TZ in line with transcriptomic data. This result highlights the importance to have large cohort of patients to limit the misinterpretation of the overall transcriptomic and proteomic data.

**Fig. 2:**
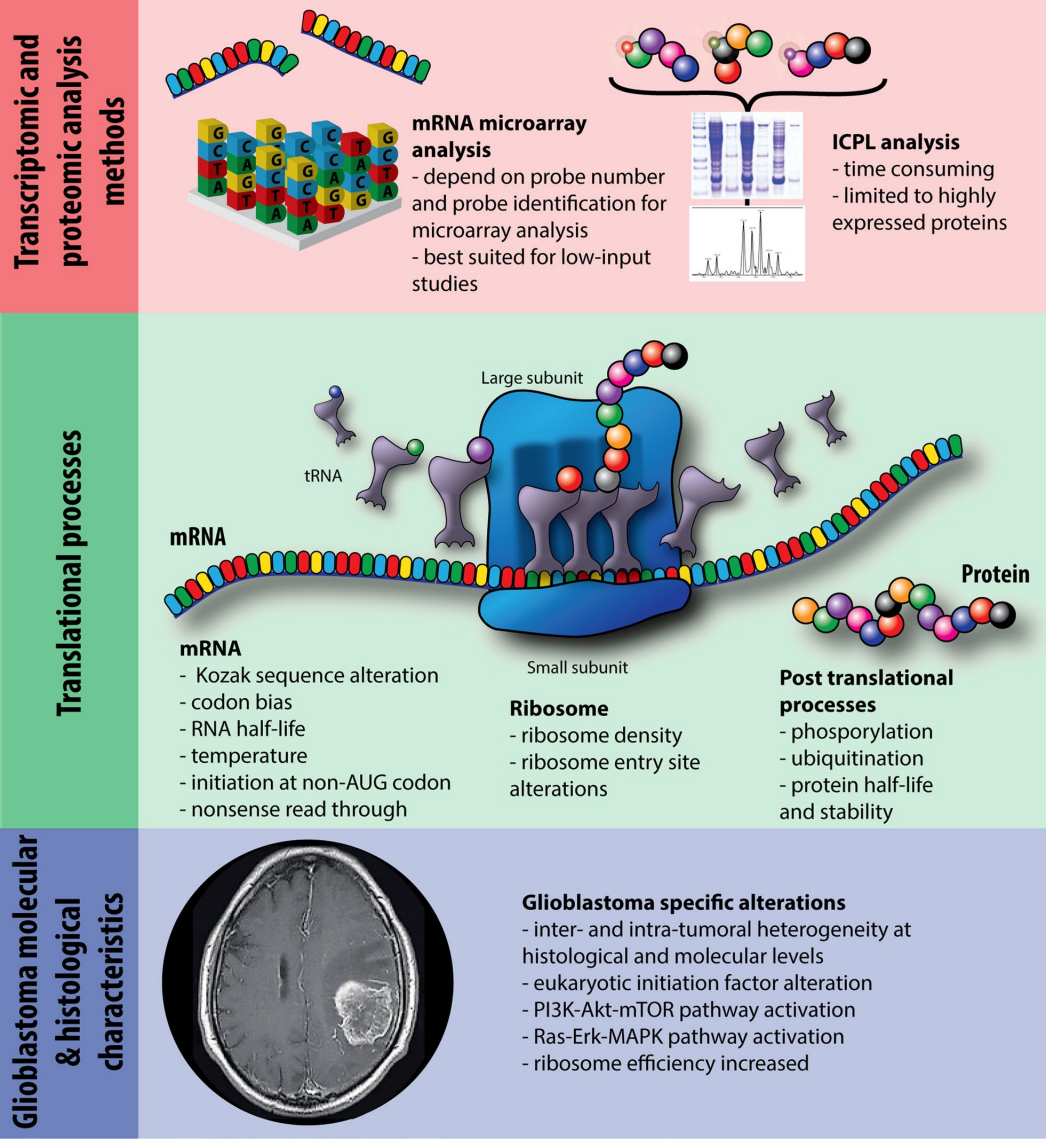
Summary of potential bias when comparing transcriptome and proteome data. This figure summarizes various mechanisms that may alter translation, and lead to differences between transcriptome and proteome (Kozak sequence: initiating sequence for translation, located on the mRNA; non-sense read through: misreading of the mRNA in the opposite direction from 3′ to 5′)

The low correlation between transcriptome and proteome data is not new and not restricted to cancer tissue [28–33]. For example, a correlation of only 17% was found between mRNAs and proteins in lung adenocarcinoma [28, 34]; in prostate cancer, the correlation between gene expression and protein levels is also poor to moderate [35]. Song et al. [36] performed proteomic profiling of eight GBs and their paired normal brain tissues and afterwards assessed overlap with RNA gene expression profiling from GEO and TCGA datasets of GBs. They found a correlation of only 2% between the differentially expressed proteins and genes from microarray. The low overlap between transcriptome and proteome data observed in our study can be explained by the general biological phenomena described above but also by analytical bias and GB-specific alterations (Fig. 2). On the analytical side, the choice of the analysis technique is crucial in omic studies and directly affects both the results and the feasibility of comparison between different datasets [37, 38]. RNA microarray techniques are the most widely used methods, allowing fast and accurate identification of mRNAs [39]; however, the number of probes on the microarray chip limits the extent of mRNA detection and probe set identification is a source of error in mRNA identification [40]. The ICPL method used here for proteome analysis allows assessment of protein levels but only a fraction of the proteome corresponding to the abundant proteins is analyzed [24]. Furthermore, only 60% of the identified proteins in one analysis were quantified. Another issue is that correlation coefficients are a crude method of measuring associations. For example, they do not account for interactions. However, because our analysis is based on only five samples it would not be meaningful to apply more advanced statistical methods. More than these analytical biases, GB tumor tissue possesses several specific properties that may influence the comparison between transcriptome and proteome data. GB is by definition an inter- and intra-heterogeneous tumor, which complicates the comparison between the two different analyses. The inter-heterogeneity is well defined at the level of the TZ through the identification of several GB subtypes. Recently, we described that this inter-heterogeneity was also present at the level of the PBZ [15, 18–20]. We identified two extratumoral microenvironments that can be encountered in the PBZ of GB patients: an extratumoral microenvironment containing GB-associated stromal cells (GASCs) with procarcinogenic properties and another containing GASCs without such properties [20]. As these cells may have their own specific signatures, this adds a level of complexity to make an inter-individual comparison between transcriptome and proteome data. Furthermore, the use of mirror samples to perform transcriptome and proteome analyses does not guarantee that the two samples are identical due to the intra-heterogeneity of GB [15, 22]. In our previous studies [16, 31], we confirmed this intratumoral heterogeneity at the transcriptomic and proteomic levels by comparing four regions of the GB (necrotic zone, TZ, interface zone between the tumor and the parenchyma and PBZ). The proteomic analysis generated a specific dataset of proteins for which a gradient of over-expression was observed from the periphery to the core of the GB [31]. At the transcriptome level, we observed that the molecular heterogeneity was much more important within tumors than between patients [16, 41]. The molecular definition of this intratumoral heterogeneity of GB is still incomplete, but the data are rapidly growing. For example, Nobusawa et al. [42] observed numerous tumor area-specific genomic imbalances, and our previous study as others reported inherent intratumor molecular subtype heterogeneity in GBs [7, 16, 43]. Other studies showed that RTK amplifications as well as MGMT status are heterogeneously distributed in GB [44–46]. More recently, next-generation sequencing techniques were able to highlight this intratumoral heterogeneity, to identify different clonal population of GB cells and understand their role in the recurrence [47, 48].

Of interest, the search of TFBSs upstream from the open reading frames encoding the deregulated proteins that may explain the modification of their level of expression revealed a common binding site for TOPORS. We observed through Western blot analysis an expression of TOPORS in 7/11 TZ samples while no expression was evidenced in PBZ. The consultation of the Human Protein Atlas is consistent with our data where a higher expression of TOPORS was observed through immunohistochemistry analysis in 5/11 glioma specimens relative to brain tissue [49]. TOPORS is a RING finger protein that was identified originally as a topoisomerase I-binding protein and as p53-binding protein. TOPORS was shown to function as both a ubiquitin and SUMO E3 ligase for p53 [50, 51]. Its overexpression leads to a proteasome-dependent decrease in p53 [50]. Human TOPORS is located on chromosome 9p21, a region found frequently altered in several different malignancies of which GBs [52]. Some findings suggest that TOPORS may function as a tumor suppressor [53, 54]; further studies are needed to clarify the exact clinical significance as well as the exact biological function of TOPORS expression in GBs.

## Conclusions

In this study, we showed a low correlation between transcriptome and proteome data for GB samples as described in other cancer tissues. We recognize that the number of studied samples was only five and that only a single sample per patient was used indicating that the results should be considered observational at this time. Future multi-omics studies must be performed on large cohort of patients and on spatially distinct tumor fragments per patient to consider the inter- and intra-heterogeneity of GBs. This may lead to an interpretation accuracy of the overall transcriptomic and proteomic data. We observed that NEFL, SYN1 and 12 biological processes were deregulated in both the transcriptome and proteome data. It will be important to analyze more specifically these processes and these two proteins to allow the identification of new theranostic markers or potential therapeutic targets for GB. Furthermore, a more detailed study of TOPORS which its TFBS was present upstream from the open reading frames of all proteins altered between TZ and PBZ may be promising.

## Additional files

**Additional file 1: Figure S1.** Graphical abstract.

**Additional file 2: Table S1.** Differentially expressed probes between TZ and PBZ pooled samples transcriptomic analysis.

**Additional file 3: Table S2.** List of altered biological processes in pooled GB transcriptomic and proteomic profiles.

**Additional file 4: Table S3.** List of identified TFBS located upstream of deregulated proteins’ coding region.

## Abbreviations

EBI: European bioinformatics institute
FDR: flase detection rate
GASC: glioma-associated stroma cells
GB: glioblastoma
ICPL: isotope-coded protein labeling
MGMT: O^6^ methyl guanine methyl transferase
MS: mass spectrometry
NEFL: neurofilament light polypeptide
PBZ: peritumoral brain zone
PVDF: polyvinylidene difluoride
RTK: receptor tyrosine kinase
SYN1: synapsin 1
TCGA: the cancer genome atlas
TFBS: transcription factor binding site
TOPORS: TOP1 binding arginine/serine rich protein
TZ: tumor zone
