# Supplementary figures and images for: Integration of transcriptome and proteome profiles in glioblastoma: looking for the missing link
Source: BMC Mol Biol. 2018 Nov 21;19:13. doi: 10.1186/s12867-018-0115-6 (PMC6249855; doi:10.1186/s12867-018-0115-6)

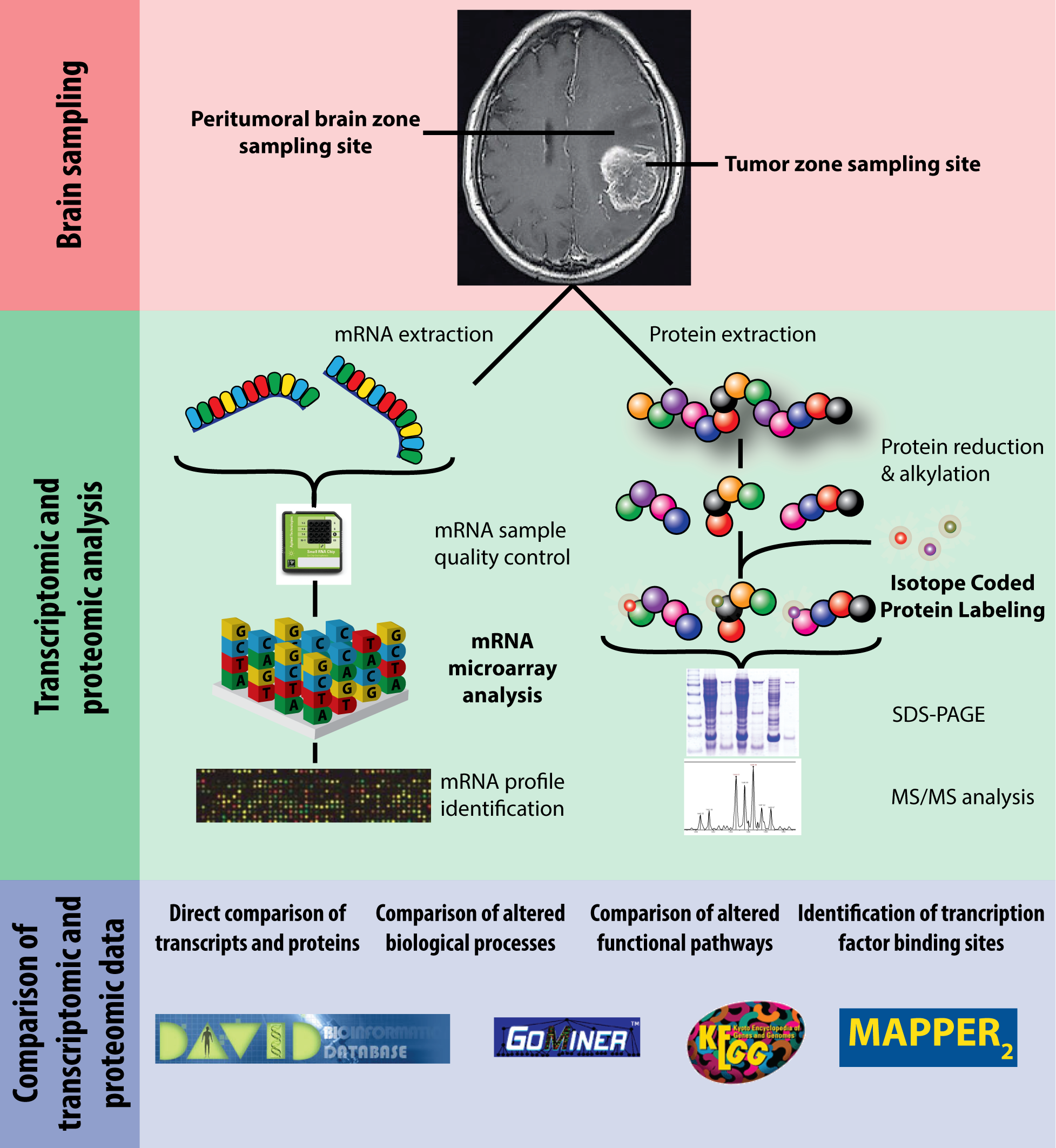

Supplement: Supplementary file 1 — Additional file 1: Figure S1. Graphical abstract. [file 12867_2018_115_MOESM1_ESM.tif]
